# Bayesian estimation of associations between identified longitudinal hormone subgroups and age at final menstrual period

**DOI:** 10.1186/s12874-015-0101-3

**Published:** 2015-12-18

**Authors:** Bei Jiang, Mary D. Sammel, Ellen W. Freeman, Naisyin Wang

**Affiliations:** Department of Mathematical and Statistical Sciences, University of Alberta, Edmonton, AB Canada; Department of Biostatistics and Epidemiology, Perelman School of Medicine, University of Pennsylvania, Philadelphia, PA USA; Departments of Obstetrics and Gynecology and Psychiatry, Perelman School of Medicine, University of Pennsylvania, Philadelphia, PA USA; Department of Statistics, University of Michigan, Ann Arbor, MI USA

**Keywords:** Menopause, Reproductive aging, Reproductive hormones, Joint modeling of longitudinal and time-to-event data, Mixture modeling, Penalized splines

## Abstract

**Background:**

Although follicle stimulating hormone (FSH) is known to be predictive of age at final menstrual period (FMP), previous methods use FSH levels measured at time points that are defined relative to the age at FMP, and hence are not useful for prospective prediction purposes in clinical settings where age at FMP is an unknown outcome. This study is aimed at assessing whether FSH trajectory feature subgroups identified relative to chronological age can be used to improve the prediction of age at FMP.

**Methods:**

We develop a Bayesian model to identify latent subgroups in longitudinal FSH trajectories, and study the relationship between subgroup membership and age at FMP. Data for our study is taken from the Penn Ovarian Aging study, 1996–2010. The proposed model utilizes mixture modeling and nonparametric smoothing methods to capture hypothesized latent subgroup features of the FSH longitudinal trajectory; and simultaneously studies the prognostic value of these latent subgroup features to predict age at FMP.

**Results:**

The analysis identified two FSH trajectory subgroups that were significantly associated with FMP age: 1) early FSH class (15 %), which displayed initial increases in FSH shortly after age 40; and 2) late FSH class (85 %), which did not have a rise in FSH until after age 45. The use of FSH subgroup memberships, along with class-specific characteristics, i.e., level and rate of FSH change at class-specific pre-specified ages, improved prediction of FMP age by 20–22 % in comparison to the prediction based on previously identified risk factors (BMI, smoking and pre-menopausal levels of anti-mullerian hormone (AMH)).

**Conclusions:**

To the best of our knowledge, this work is the first in the area to demonstrate the existence of subgroups in FSH trajectory patterns relative to chronological age and the fact that such a subgroup membership possesses prediction power for age at FMP. Earlier ages at FMP were found in a subgroup of women with rise in FSH levels commencing shortly after age 40, in comparison to women who did not exhibit an increase in FSH until after 45 years of age. Periodic evaluations of FSH in these age ranges are potentially useful for predicting age at FMP.

**Electronic supplementary material:**

The online version of this article (doi:10.1186/s12874-015-0101-3) contains supplementary material, which is available to authorized users.

## Background

Information about age at natural menopause is important for counseling women about reproductive planning and for treating women who experience hormone-sensitive gynecological conditions such as endometriosis and fibroids. Moreover, predicting age at menopause may help determine risks for hormone-related adverse health outcomes, such as breast cancer, endometrial cancer, osteoporosis and cardiovascular disease [1]. Prospective prediction of age at final menstrual period (FMP) would be beneficial in treatment planning as accelerated bone lose begins one or more years prior to the FMP [2, 3]. Researchers have previously evaluated detailed menstrual diaries [4] and chronologic age to predict FMP. More recently, interest has switched to examining reproductive hormone levels, including follicle stimulating hormone (FSH), estradiol (E2) and anti-mullerian hormone (AMH) as predictors of FMP [2, 5, 6].

An objective of this study was to describe salient features of prospectively collected FSH levels during the menopause transition and identify features that are associated with age at final menstrual period (FMP). Previous studies [5–7] utilized classic Cox Proportional Hazard models to assess the impact of reproductive hormones and time to menopause based on a one-time assessment of risk factors. Other descriptive studies aligned time to the date of FMP and described patterns of menstrual cycle lengths [5] or longitudinal hormone trajectories relative to FMP [8, 9]. Greendale et al. [2] used longitudinal FSH levels to predict time to menopause by selecting observed hormone levels at specific points in time proximal to the FMP, i.e. 2 years prior, 1 year prior, etc. Although informative, this method is not practical for prospective prediction of the FMP in clinical practice.

Our proposed approach differs from these previous studies in several ways. First, we describe the longitudinal FSH trajectory patterns in relation to chronological age, and allow the inclusion of women who had not yet reached their FMP, and employed a generalized growth mixture model (GGMM) [10] to identify informative FSH subgroups in relation to age. We then utilized the combinations of the resulting individual’s subgroup or class membership and class-specific characteristics (level and rate of FSH change at class-specific pre-specified ages) to estimate associations with FMP age under a Bayesian accelerated failure time (AFT) model. This approach accounted for uncertainty in the estimation of FSH trajectory features thereby increasing statistical power.

## Methods

### POAS database

The study evaluated 363 of the original 436 women from the Penn Ovarian Aging Study (POAS). The POAS cohort was identified through random-digit dialing to households in Philadelphia County, PA in 1996, with stratified sampling to obtain equal numbers of African American and Caucasian women, as described previously [11]. The subset in our analysis consisted of women who were followed to, and did not reach, FMP until after age 40, had AMH assessed at baseline, recorded values for the demographic covariates (BMI, race and smoking) and had at least 6 hormone measurements over the study period. All participants provided written informed consent, and the Institutional Review Board of the University of Pennsylvania approved the study.

At enrollment, all participants were premenopausal as defined by regular menstrual cycles in the reference range (22–35 days for the previous three menstrual cycles), were ages 35–48 years with an intact uterus and at least one ovary. Exclusion criteria included current use of psychotropic or hormonal medications, including hormonal contraception and hormone therapies, pregnancy or breast feeding, serious health problems known to compromise ovarian function (e.g., diabetes mellitus, liver disease, breast or endometrial cancer) and alcohol or drug abuse in the past year.

The cohort was followed for 14 years after enrollment. Follow-up assessments were at approximately 9-month intervals for the first 5 years and then annually. At each assessment, there were 2 in-home visits to collect study data and blood samples for the hormone assays. All visits were timed to the early follicular phase (days 2–6) of the menstrual cycle and were conducted in two consecutive menstrual cycles or approximately one month apart in non-cycling women.

The study was described to participants as a general women’s health study. At each assessment, trained research interviewers obtained structured interview data on overall health including menstrual cycle information, blood samples for the hormone assays, and anthropometric measures (height, weight, waist and hip circumference).

### Study variables

The primary outcome variable was age at FMP. Age at FMP was calculated from her reported age at the first follow-up assessment where the participant reported no menstrual bleeding for at least 12 months.

Hormone values were assayed from blood samples that were obtained at each study visit (days 2–6 of the menstrual cycle), centrifuged and frozen in aliquots at −80 C. FSH and E2 were measured by radioimmunoassay in the Clinical Translational Research Center (CTRC) of the University of Pennsylvania using Coat-A-Count commercial kits (Siemens). Inter-assay and intra-assay coefficients of variation were less than 5 %. AMH measures from the first available frozen samples in assessments 1–3 were assayed contemporaneously in 2011 in the CTRC of the University of Pennsylvania, using AMH enzyme-linked immunosorbent assay kits (Beckman Coulter Inc., Brea, CA). The intra- and inter-assay coefficients of variation were 4.6 and 6.8 %, respectively. The lower limit of detection was 0.10 ng/mL. Hormone measurements when women were pregnant and/or breast feeding or had a hysterectomy with or without oophorectomy were not included in the analysis.

Other covariates that were selected as possible risk factors for FMP age included race (African American or Caucasian), body mass index (kg/ *m*^2^) adjusted to age 40 (defined below), and baseline smoking status (yes, no) [11].

AMH and BMI values were adjusted to age 40 values for all participants. This was accomplished by fitting local polynomials using the lprq function in R package “quantreg” to the median of AMH and BMI respectively versus age to obtain predicted AMH and BMI values at age 40. This age was selected a priori as an appropriate reference age for prediction of FMP, and because AMH levels decrease rapidly to non-detectible levels in this age range [11, 12], resulting in insufficient numbers of observed AMH values in the detectible range to facilitate longitudinal modeling of this hormone.

### Statistical analysis

We proposed a Bayesian joint model for the FSH trajectories and FMP age to properly account for uncertainty in estimating FSH trajectory features and thus gain statistical efficiency by reducing the bias towards the null hypothesis of no association [13]. It consists of defining a longitudinal model for the FSH trajectories and defining a primary outcome model for the FMP age using the extracted FSH features as covariates. The joint modeling refers to methods to analyze the longitudinal model and the primary outcome model jointly or simultaneously [14, 15]. For the FSH trajectories, we utilized a GGMM [10] to identify distinct feature subgroups (i.e., latent classes); details of the GGMM are given in Additional file 1. In the GGMM, we used cubic Bayesian penalized splines [16] to flexibly model FSH levels evaluated at unequally spaced times, where the rate of change is represented as the first derivative of the smoothed mean FSH level, and we assumed a t-distribution with 4 degrees of freedom for the model residuals [17] to accommodate large fluctuations in FSH levels. In addition, we modeled FSH within-subject variability using a lognormal distribution [10, 18] and studied its predictive ability towards FMP age. The FSH within-subject variability contributed to increased risk of severe hot flashes [10]. Our approach naturally accommodates the fact that the subject-level mean hormone trajectories may differ from one another and may be grouped into classes characterized by similar trajectory shapes. In contrast to fully parametric splines, the use of penalized splines is not as sensitive to the exact number and location of the knots. This added flexibility is achieved as long as a sufficiently large number of knots are used so that redundant knots will be smoothed away by shrinking associated random effects toward 0. The use of a heavier tailed t-distribution allows robust inference by avoiding the potential influence of outlying hormone levels.

For the outcome model to predict FMP age, we employed accelerated failure time (AFT) models [19, 20]. We define the following notation. Let *T*_*i*_ denote age at FMP for unit *i*, *D*_*i*_ denotes which FSH trajectory subgroup the unit belongs to; *μ*_*i*_(*τ*) is the mean FSH hormone level at age *τ*, *ν*_*i*_(*τ*) is the rate of change in FSH hormone levels at age *τ*, and *σ*_*i*_^2^ is the within-subject variability that captures the short-term fluctuations in FSH hormone levels. In particular, we assume a lognormal AFT model for *T*_*i*_ conditional on covariates of interest. This is equivalent to assuming the residual in the AFT model has a normal distribution, i.e., *ε*_*i*_ ∼ *N*(0, *σ*^2^). We found no violation of this assumption when Cox-Snell residuals were evaluated. We consider various features extracted from the GGMM model defined for the FSH trajectories as covariates of AFT models to predict FMP ages (Models *M*_*s*_, s = 1... 4 in Table 1). Each of these AFT models was jointly estimated with the GGMM model for the FSH trajectories. We also compare these joint models with a baseline AFT model (Model *M*_0_ in Table 1) that does not include FSH feature as covariates.

**Table 1:** Specification of the AFT models to predict ages at final menstrual period (*T*_*i*_)

Model	AFT models
*M*_0_	log(*T*_*i*_ ‐ 40) = *α*_0_ + *x*_*i*_^*T*^ *θ* + *ε*_*i*_
*M*_1_	log(*T*_*i*_ ‐ 40) = *α*_0_ + *α*_1_ *D*_*i*_ + *α*_2_ *ω*_*i*_ + *x*_*i*_^*T*^ *θ* + *ε*_*i*_
*M*_2_	log(*T*_*i*_ ‐ 40) = *α*_0_ + *α*_1_ *D*_*i*_ + *α*_2_ *ω*_*i*_ + *α*_3_ *μ*_*i*_(40)(1 ‐ *D*_*i*_) + *α*_4_ *μ*_*i*_(45)*D*_*i*_ + x_*i*_^*T*^ *θ* + *ε*_*i*_
*M*_3_	log(*T*_*i*_ ‐ 40) = *α*_0_ + *α*_1_ *D*_*i*_ + *α*_2_ *ω*_*i*_ + *α*_3_ *ν*_*i*_(40)(1 ‐ *D*_*i*_) + *α*_4_ *ν*_*i*_(45)*D*_*i*_ + x_*i*_^*T*^ *θ* + *ε*_*i*_
*M*_4_	log(*T*_*i*_ ‐ 40) = *α*_0_ + *α*_1_ *D*_*i*_ + *α*_2_ *ω*_*i*_ + *α*_3_ *μ*_*i*_(40)(1 ‐ *D*_*i*_) + *α*_4_ *μ*_*i*_(45)*D*_*i*_ + *α*_5_ *ν*_*i*_(40)(1 ‐ *D*_*i*_) + *α*_6_ *ν*_*i*_(45)*D*_*i*_ + x_*i*_^*T*^ *θ* + *ε*_*i*_

*Note:*

● *x*_*i*_^*T*^ = (adjusted log (BMI) at age 40, adjusted AMH at age 40, smoking, race)

● *ε*_*i*_ ∼ *N*(0, *σ* ^2^) is the residual for log-normal AFT model

● *D*_*i*_ is the early FSH rise class indicator and *ω*_*i*_ is the within-subject variability in FSH; *μ*_*i*_(*τ*) and *ν*_*i*_(*τ*) are the mean FSH level and its rate of change at age *τ*, respectively

Because of possible censoring due to not reaching FMP by the end of the study, hysterectomy or dropout, FMP was not always observed. For these women we observe age at last visit or censoring age, *C*_*i*_. Assuming independence between censoring time and FMP age, we imputed FMP age, *T*_*i*_, based on the specific AFT model defined in Table 1. The imputed value was a random draw from the conditional posterior distribution, a truncated normal distribution with its mean determined by the AFT model conditional on other covariates and variance *σ*^2^ [21]. Model predictive performance or model fit, was assessed using prediction Mean Squared Error (PMSE) estimated from a 10-fold cross-validation [22] for measured FMP age. Statistical significance of covariate effects was determined when the 95 % Bayesian Credible Interval (CI) did not contain 0.

We develop an efficient Markov chain Monte Carlo algorithm (MCMC) for posterior sampling with equivalent prior specifications as used in [10] (Additional files 2 and 3). The inferences were based on 25,000 posterior draws after a burn-in period of 10,000 iterations. All the computations were performed by calling stand-alone C++ code in R software developed using the Scythe statistical C++ library [23].

## Results

Out of the 363 women in the analysis, 180 reported the FMP during the follow-up period. Of the remaining participants, 157 did not reach their FMP and were considered censored at their last assessment, and 26 had a hysterectomy in the follow-up period before reaching FMP and were considered censored at the time of hysterectomy.

**Fig. 1:**
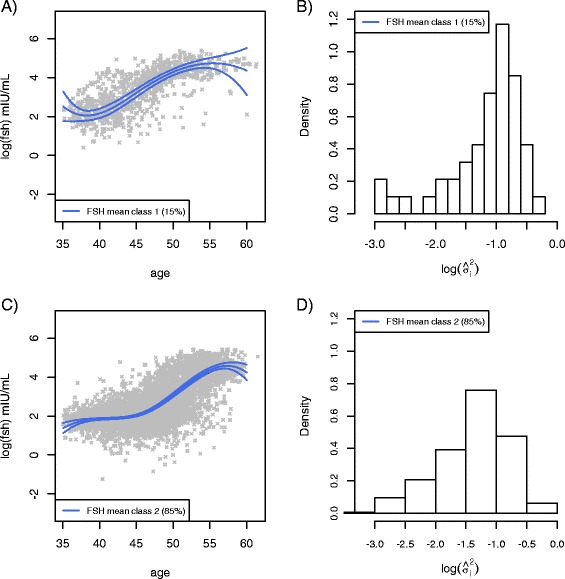
Fitted mean FSH trajectories from model *M*_2_ for early FSH rise (FSH class 1, Panel 1.**a**) and late FSH rise (FSH class 2, Panel 1.**c**), along with a histogram of the log-transformed estimated within subject variance, *σ*_*i*_^2^ for each class (Panels 1.**b** and 1.**d**).

Two latent classes for mean FSH trajectories were identified within the GGMM by Deviance Information Criteria [24, 25], where D_i_ = 0 is defined as class 1, early riser of FSH and *D*_*i*_ = 1 is class 2, late riser of FSH. Figure 1a and c show the estimated mean FSH trajectory over time for each class. Class 1 consists of women who tend to have an earlier rise of FSH (15 % of the sample), and class 2, women who tend to have a late rise of FSH in their mid-40s (85 % of the sample).

Characteristics of the participants are summarized in Table 1. The mean (SD) age of these participants was 43.1 (3.1) yr at baseline and 50.8 (3.2) yr at FMP. The mean (SD) of FSH levels at baseline was 8.71 (4.88) mIU/mL. The mean (SD) of adjusted AMH levels at age 40 was 0.94 (0.77) ng/mL. These adjusted AMH levels were further divided into quartiles to better understand the association with FMP age. Table 1 also shows the comparison of 47 women who were classified as class 1 with the 316 women assigned to class 2. In addition to differences in E2 and FSH, women in class 1 had lower adjusted BMI (*P* < 0.05) and lower AMH levels (*P* < 0.001). There were no differences in race (*P* = 0.565). Although women in class 1 were more likely to smoke, this comparison did not achieve statistical significance (*P* = 0.063).

Table 2, column 2 describes the associations between FMP age and baseline risk factors. BMI and AMH at age 40 were positively associated with FMP age, i.e., women with higher BMI have an older FMP age. Similarly, a higher adjusted AMH at age 40 (>0.54 ng/mL, 1st quartile) is associated with an older FMP age. As expected, smoking resulted in an earlier FMP age.

**Table 2:** Participant characteristics and comparisons between FSH early and late rise classes

	Total	Early rise class	Late rise class	
	*N* = 363	*N* = 47	*N* = 316	*P* value
Age at baseline, mean (SD)	41.62 (3.43)	40 .67 (3.65)	41.67 (3.38)	0.060
Race, N (%)				0.565
Caucasian	188 (52)	22 (53)	166 (53)	
Af. American	175 (48)	25 (47)	150 (47)	
Smoker at baseline, N (%)	137 (38)	24 (51)	113 (36)	0.063
Adj BMI at age 40, mean (SD)	28.42 (7.85)	26.07 (7.04)	28.77 (7.92)	0.013
Adj. AMH at age 40 (ng/mL), mean (SD)	1.17 (1.11)	0.39 (0.59)	1.29 (1.12)	<0.001
AMH quartile, N (%)				<0.001
Q1: <=0.54 ng/mL	91 (25)	29 (62)	62 (20)	
Q2: >0.54 & < =0.83 ng/mL	91 (25)	12 (26)	79 (25)	
Q3: >0.83 & < =1.51 ng/mL	90 (25)	4 (9)	86 (27)	
Q4: >1.51 ng/mL	91 (25)	2 (4)	89 (28)	
Baseline FSH mIU/mL, mean (SD)	7.92 (4.69)	12.03 (7.97)	7.31 (3.62)	<0.001

The results for models that consider FSH trajectory subgroups are presented in Table 3 columns 3–6. Women in class 2 reached FMP at later ages in comparison to women in class 1 (model *M*_1_ (column 3, Table 3). On average, it took 1.53 (exponentiations of the effect of being in class 2 in lognormal AFT model *M*_1_) (95 % CI: 1.33, 1.77) times additional years post age 40 to reach FMP for women in class 2 compared to women in class 1. Adding such FSH trajectory class memberships improved model fit over the model containing only AMH and demographics covariates. Model *M*_2_ and *M*_3_ (Table 3) took into account different timing of FSH rise for women in early and later FSH rise classes as well as potentially additional contributions from FSH levels and rates of change at age 40 and 45 respectively. The use of alternative ages (e.g., 41 and 46 years) in each FSH subgroup resulted in similar outcomes (not shown).

**Table 3:** Coefficient estimates (95 % credible interval) for all candidate models

	*M*_0_ (Column 2)		*M*_1_ (Column 3)		*M*_2_ (Column 4)		*M*_3_ (Column 5)		*M*_4_ (Column 6)	
	Value	95 % CI	Value	95 % CI	Value	95 % CI	Value	95 % CI	Value	95 % CI
**Class 2 vs 1**			**0.424**	**0.288, 0.570**	0.077	−0.568, 0.695	**0.406**	**0.135, 0.677**	−0.109	−0.814, 0.580
**Class 1:** *μ*_*i*_(40)					**−0.442**	**−0.685, −0.213**			**−0.420**	**−0.671, −0.175**
**Class 2:** *μ*_*i*_(45)					**−0.273**	**−0.460, −0.096**			−0.167	−0.411, 0.087
**Class 1: ν**_*i*_(40)							**−1.185**	**−2.873, 0.619**	−0.624	−2.327, 1.120
**Class 2: ν**_*i*_(45)							**−0.850**	**−1.486, −0.236**	−0.517	−1.365, 0.346
**Within women Variability**			−0.515	−1.289, 0.247	−0.011	−0.132, 0.108	−0.021	−0.149, 0.101	−0.008	−0.130, 0.108
**Intercept**	1.376	0.672, 2.090	1.777	1.099, 2.341	2.790	1.985, 3.614	1.895	1.196, 2.509	2.810	2.019, 3.591
**Adj log (BMI)**	**0.240**	**0.043, 0.437**	0.073	−0.078, 0.249	0.044	−0.127, 0.208	0.059	−0.093, 0.234	0.044	−0.098, 0.207
**Adj AMH**_**40**_ **2** ^**nd**^ **Q**	**0.249**	**0.116, 0.381**	**0.151**	**0.026, 0.277**	0.089	−0.038, 0.213	0.122	−0.005, 0.250	0.079	−0.042, 0.202
**Adj AMH**_**40**_ **3rd Q**	**0.421**	**0.281, 0.556**	**0.274**	**0.137, 0.407**	**0.179**	**0.046, 0.310**	**0.219**	**0.081, 0.363**	**0.163**	**0.032, 0.296**
**Adj AMH**_**40**_ **4th Q**	**0.420**	**0.274, 0.567**	**0.241**	**0.088, 0.391**	**0.155**	**0.008, 0.300**	**0.184**	**0.037, 0.331**	0.137	−0.005, 0.280
**Smoking**	**−0.162**	**−0.263, −0.058**	**−0.138**	**−0.234, −0.040**	**−0.116**	**−0.206, −0.029**	**−0.130**	**−0.225, −0.038**	**−0.111**	**−0.201, −0.022**
**Race (Caucasian)**	0.059	−0.039, 0.155	0.030	−0.057, 0.123	0.006	−0.082, 0.093	0.029	−0.061, 0.117	0.008	−0.077, 0.094
**Root PMSE**	3.26	2.45, 4.29	2.89	2.28, 3.91	2.67	1.89, 3.53	2.76	2.03, 3.67	2.66	1.84, 3.47

Note: significant coefficients at the 0.05 level are in bold face.

The model with best fit, determined by minimum PMSE, was model *M*_4_ (the model which included all predictor variables of interest). However, adding both FSH levels and rates of change at age 40 and 45 for early and later FSH rise classes respectively minimally improved the PMSE over model *M*_2_ (Table 3, column 4). Also, the parameters associated with the rate of change in FSH at age 40 or 45 were not statistically significant in model *M*_4_. Therefore, the simpler model *M*_2_, with root PMSE = 2.67, was selected as the best model to describe the associations between FSH and FMP age.

In the model *M*_2_, for the 15 % of women assigned to early rise FSH class, the FMP age was inversely associated with their FSH level at age 40 and for the remaining 85 % of women whose FSH began to increase in their mid-40s, the association between FMP age was inversely associated with their FSH at age 45. In this model, smoking significantly decreased the FMP age, while higher AMH levels at age 40 were associated with later FMP age. Short-term variability in FSH residuals was not associated with FMP age. Neither BMI nor race was significantly associated with FMP age after accounting for the other risk factors.

**Fig. 2:**
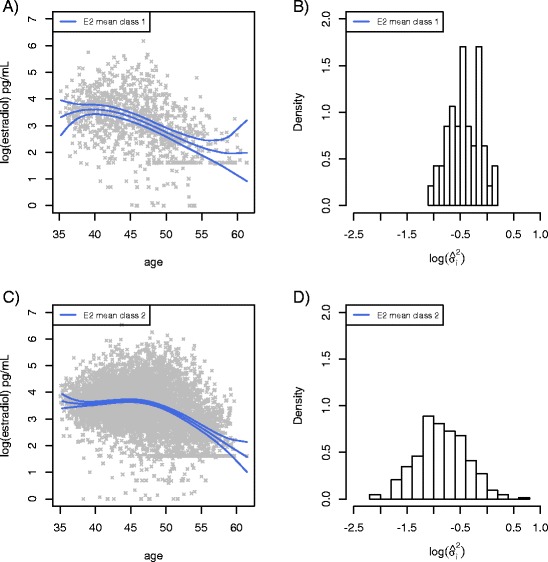
Fitted mean Estradiol trajectories for early FSH rise (FSH class 1, panel 2.**a**) and late FSH rise (FSH class 2, panel 2.**c**), along with log-transformed estimated within subject variance, σ^2^ for each class(panels 2.**b**B and 2.**d**). Significant differences in the estimated variances of estradiol indicated were detected with two-sample *t*-test (*P* < 0.001)

E2 trajectories were examined, but the current modeling method did not allow identification of meaningful subclasses for E2 trajectories. Consequently, we applied the class structure derived from the GGMM of the FSH trajectories, and examined E2 trajectories according to this class. Figure 2a and c illustrate the decline in E2 for each subgroup. Class specific within-subject variability in E2 shown in Fig. 2b and d, suggested that women in class 1, whose FSH levels increased and E2 decreased at younger ages had relatively larger variability in E2 values than women in class 2, whose FSH levels increased and E2 decreased at older ages. A two-sample *t*-test of the estimated variances of E2 indicated a significant difference (*P* < 0.001); the difference in the estimated variances of FSH is marginally significant (*P* = .052). These findings further suggest the potential for physiological differences between the women in the two identified subgroups. In contrast, significant differences in the estimated variances for the two FSH classes do not seem to exist, as shown in Fig. 1b and d, where the distributions of the estimated variances have similar spread.

**Fig. 3:**
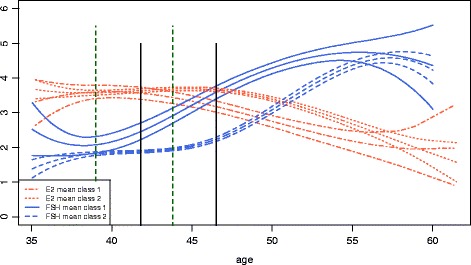
Fitted mean FSH trajectories (*blue*) for early FSH rise (FSH class 1) and late FSH rise (FSH class 2) from model *M*_2_; and corresponding Estradiol mean trajectories (*orange*) for these two FSH classes. Green vertical bar indicates begin of the rise in FSH. Black vertical bar indicates initiation of Estradiol decline

Figure 3 overlays the fitted FSH and E2 trajectories for class 1 (solid lines), and 2 (dashed lines). For class 1, the corresponding decline in E2 appears to be delayed relative to the initial increase in FSH when compared to the behavior in class 2. The closeness in distance of two E2 mean trajectories also explains the difficulties in identifying the two subgroups using E2.

Additional analyses were conducted to examine the performance of a model that included all women with FSH levels at age 40 or 45 years, rather than the FSH trajectory subgroups, and also included AMH and demographic covariates. In this model, adding FSH level at age 40 did not improve model fit over and above a model with AMH and demographic covariates alone (root PMSE = 3.21, 95 % CI: 2.47, 4.08, estimates not shown). In contrast, adding FSH level at age 45 did improve prediction of FMP age based on AMH and demographic covariates alone (root PMSE = 2.75, 95 % CI: 1.98, 3.52), perhaps because FSH level at age 45 served as a surrogate for the FSH classes. While improved over a model of risk factors alone, this model was inferior to our best models M_2_ and *M*_4_. Another model, which added the E2 level or its rate of change at age 45 and variability in residuals, did not improve the model fit over model *M*_0_ with root PMSE = 3.27, 95 % CI: 2.49, 4.26 and root PMSE = 3.26, 95 % CI: 2.51, 4.25, respectively (estimates not shown).

**Fig. 4:**
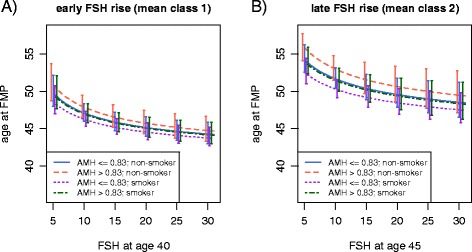
Predicted age at FMP with 95 % CI for early FSH rise (FSH class 1) and late FSH rise (FSH class 2) based on our final model

To effectively predict age at FMP, we removed insignificant covariates and simplified our best models, M_2_ and M_4_, to the following final prediction model, where BMI, race, within-subject variability in FSH were dropped from *M*_2_ and quartiles of AMH were collapsed to 2 levels (AMH < = 0.83 and AMH > 0.83); other terms in *M*_*2*_ were unchanged. Figure 4a and b show the predicted FMP age at different FSH levels for each class, using this model for 4 sub-categories defined by the two levels of AMH and smoker/non-smoker. Separate plots are displayed for early and late rise FSH classes respectively. For example, for early risers with FSH level of 10 at age 40, the predicted ages at FMP for non-smokers with AMH < = 0.83 and smokers with AMH > 0.83 are 47.1 year, 95 % CI: 46.0, 48.3 and 47.0 year, 95 % CI: 45.8, 48.3 respectively; for late risers with FSH level of 10 at age 45, the results change to 51.7 years, 95 % CI: 50.4, 53.1 and 51.5 years, 95 % CI: 50.0, 53.1 respectively. This figure clearly illustrates the effectiveness of using the FSH class memberships to predict FMP age (i.e., non-overlapping of the 95 % credible intervals for each FSH class), while the contribution due to AMH and smoking were not as strong, indicated by the considerable overlap in the 95 % credible intervals at various values of FSH. This graph suggests that the impact of smoking on FMP age is similar to that of AMH above/below the median value of 0.83 ng/mL.

## Discussion

In this study, we demonstrated that our model of prospectively collected longitudinal measurements of FSH identified 2 subgroups of women with distinct FSH trajectories that were significantly associated with FMP age. The first subgroup, comprising 15 % of the study sample, displayed initial increases in FSH shortly after age 40, while the second subgroup (85 % of the sample) did not have a rise in FSH until after age 45. We then showed that class-dependent FSH values, at age 40 or 45, were significantly associated with FMP age. Importantly, these associations remained significant after adjustment for AMH, BMI and smoking, which are established risk factors for earlier age at menopause. We also found that neither the rate of increase nor the within-woman variability in FSH was associated with FMP age. To the best of our knowledge, our work is the first in this area to show the existence of latent classes in FSH trajectory patterns relative to chronological age.

Using the same analytic approach for estradiol showed that there was no heterogeneity in the longitudinal pattern of this hormone in the present study. However, when FSH class structure was applied to E2, we identified distinct profile differences, with significant increases in E2 variability in the subgroup who had early FSH rise (class 1) compared to the subgroup with FSH rise after age 45 (class 2). These results suggest distinct difference in physiology in the 2 subgroups beyond their FSH trajectories.

We previously predicted FMP age based on AMH from a Cox model which controlled for demographic covariates including smoking, body mass index (BMI) and race [5], while the present study identified a richer set of characteristics describing longitudinal FSH levels that contribute to the prediction of FMP age. Furthermore, this information indicated that the inclusion of the latent class memberships in various models (e.g. M_2_ and M_3_) could decrease the prediction errors by 20-22 % (based on percentage reductions in PMSE’s), indicating improved model fit. These are new findings, which we believe open a door to evaluation of reproductive hormones at specific ages to aid in the prediction of FMP age.

The findings add further information to previous studies of FMP age. In the Study of Women Across the Nation (SWAN), researchers described the pattern of reproductive hormones where the time axis was scaled to align to the date of FMP as time 0 [8]. This showed that a rise in log (FSH) began more than 6 years prior to the FMP, while the acute decline in E2 did not commence until around 2 years prior to the FMP. Similar profiles were reported in the current analysis, but our two latent classes distinguished the initial age when these changes occurred.

More recent SWAN results [9] investigated clustering of FSH and E2 trajectories pre- and post FMP age by re-centering the trajectories as 0 at FMP age, while our approach used each woman’s chronological age between 35 and 60. Tepper et al. [9] identified 3 distinct FSH classes, possibly due to the much larger sample size (*n* = 1316). The FSH levels from the three classes all tended to rise about 2 years before the FMP, and therefore these FSH classes did not differ in the timing of the rise in FSH levels. However, as implied in our analysis that rising FSH levels at an earlier age is associated with an earlier age at FMP, it is not clear whether the FSH classes obtained by the approach of Tepper et al. [9] is capable of predicting age at FMP. While both approaches utilized growth mixture models to identify subgroups of individuals sharing similar hormone trajectory patterns over time, our approach integrated additional statistical concepts (including robust inference and semi-parametric smoothing methods), modeled within-woman variability and studied its association with FMP age. More importantly, our approach utilized all available women in the analysis and classified them into the two classes with a rise in FSH levels at age 40 and 45 respectively, rather than excluding women who had not yet reached their FMP. Further, we not only evaluated the association between class membership and FMP age, our primary outcome of interest, but could also examine associations between other features of the FSH trajectories and FMP age.

Our findings were based on multiple FSH values measured over a 14-year time frame (6 to 28 observations per participant), which is generally not feasible in clinical practice. However, our findings indicate the importance of age-specific time points, suggesting that fewer annual measures at key ages, e.g., shortly after age 40 and again around age 45 if there was no FSH rise at earlier ages, would be reasonable for clinical predictions of FMP age.

An important issue in this research that has not been disentangled is the interplay between women specific risk factors, hormone changes and FMP age. Randolph and colleagues [8] reported that obese women had an attenuated FSH rate of rise in the time period prior to FMP, which would imply that FSH is an intermediate variable in the relationship between obesity and FMP age. Our results are consistent with this possibility inasmuch as BMI was associated with older FMP age in the baseline model, M_0_. However, BMI was not significantly associated with FMP age in models that included FSH class membership, FSH levels or FSH rate of change, and further studies are needed to examine the mediation effect of FSH in the association between BMI and FMP age.

## Conclusions

This paper shows that the use of the identified latent FSH trajectory features improves the prediction of time to FMP by 20 % or more (i.e., percentage reductions in PMSE’s) in comparison to the model based on AMH levels and other baseline risk factors, although AMH has been recognized as a stronger predictor for time to FMP than FSH when only one hormone is considered in each prediction model. Our proposed method quantifies time using chronological age, and also accounts for information collected from subjects who have not yet reached their FMP. In contrast, existing methods align FSH trajectories by centering each subject’s measurement time at the FMP age and require observed FMP ages for all subjects.

## Additional file

Additional file 1: Details of GGMM. (PDF 75.4 kb) Additional file 2: Specifications of the prior distributions. (PDF 81.7 kb) Additional file 3: Posterior computations through Gibbs sampling. (PDF 100 kb)
