# Supplementary material for: Bayesian estimation of associations between identified longitudinal hormone subgroups and age at final menstrual period
Source: BMC Med Res Methodol. 2015 Dec 18;15:106. doi: 10.1186/s12874-015-0101-3 (PMC4683774; doi:10.1186/s12874-015-0101-3)
Supplement: Additional file 1: — Details of GGMM. (PDF 75.4 kb) [file 12874_2015_101_MOESM1_ESM.pdf]

Details of GGMM

The GGMM for the FSH trajectories is given by:

$$
\begin{aligned}
&Y_{ij}|\boldsymbol{b}_i = \mu_i(\boldsymbol{b}_i;t_{ij}) + \varepsilon_{ij} \; e_{ij} \sim \mathrm{t}_\nu(0,\sigma_i^2) \\
&\text{or equivalently } e_{ij} \sim \mathrm{N}(0,\sigma_i^2/m_{ij}) \; m_{ij} \sim \mathrm{Gamma}(\nu/2,\nu/2) \\
&\mu_i(\boldsymbol{b}_i;t_{ij}) = \sum_{l=1}^{L} b_{il}\phi_l(t_{ij})
\end{aligned}
\tag{1}
$$

where Y_{ij} denotes FSH levels for subject i, $i = 1,...,n$ at time t_{ij}, $j = 1,...,n_i$, $\mu_{ij} \equiv \mu_i(\boldsymbol{b}_i;t_{ij})$ denotes the mean of Y_{ij} at time t_{ij} and the vector $\boldsymbol{\mu}_i = (\boldsymbol{\mu}_{i1},...,\boldsymbol{\mu}_{in_i})^T$ defines the mean profile or trajectory for subject i, where $\boldsymbol{b}_i = (b_{i1},..,b_{iL})$ is the vector of the random effects that reflects the subject-level trajectory patterns, and $\phi_l(t_{ij})$, $l = 1,...,L$ are the B spline basis functions given known knots. The t-distribution with ν degree of freedom and subject-level scale σ_i^2 is denoted by $\mathrm{t}_\nu(0,\sigma_i^2)$. To allow for "heterogeneity" in the mean profile, we consider a finite mixture of normal distributions for the random effect $\boldsymbol{b}_i$,

$$
\begin{aligned}
&D_i \sim \mathrm{Multinomial}(\pi_1^D,...,\pi_{K_D}^D); \\
&\boldsymbol{b}_i = (b_{i1},...,b_{iL})^T|D_i = d \sim \mathrm{N}(\boldsymbol{\beta}_d,\boldsymbol{\Sigma}_d), d = 1,...,K_D.
\end{aligned}
\tag{2}
$$

where, D_i defines the corresponding latent class membership for the mean profile class and $\boldsymbol{\beta}_d = (\beta_{d1},...,\beta_{dL})^T$. Thus, the fixed effect coefficients β_{dl}, $l = 1,\cdots,L$ determine the shape and also the smoothness of the mean profile for the dth latent class; the random coefficients b_{il}, $l = 1,\cdots,L$ then capture the individual deviations from the class specific mean profile. Further, for the within-subject variability parameter σ_i^2, we assume a log normal distribution $\sigma_i^2 \sim \text{log-N}(\mu,\tau^2)$.
