# Supplementary material for: Bayesian estimation of associations between identified longitudinal hormone subgroups and age at final menstrual period
Source: BMC Med Res Methodol. 2015 Dec 18;15:106. doi: 10.1186/s12874-015-0101-3 (PMC4683774; doi:10.1186/s12874-015-0101-3)
Supplement: Additional file 2: — Specifications of the prior distributions. (PDF 81.7 kb) [file 12874_2015_101_MOESM2_ESM.pdf]

Specifications of the prior distributions

We propose a fully Bayesian approach to estimate model parameters with their prior distributions given below:

- In the GGMM for FSH trajectory:
  - $\beta_{dl} \sim \mathrm{N}(\beta_{d,l-1}, \tau^2_{\beta d})$, $l = 2, ..., L$ with diffuse prior $\beta_{d1} \sim \mathrm{N}(0, 100)$ for the initial coefficient, and $\tau^2_{\beta d} \sim \mathrm{IG}(1, 0.005)$ to control the smoothness of the fitted curves.
  - $\boldsymbol{\Sigma}_d \sim$ Inverse-Wishart$(\mathrm{df} = r, \boldsymbol{\Lambda})$, where $\boldsymbol{\Lambda} = r\left(\sum_{i=1}^{n} \widehat{\mathrm{Cov}}(\tilde{\boldsymbol{b}}_i)^{-1}/n\right)^{-1}$, where $\tilde{\boldsymbol{b}}_i$ is given by OLS estimator of $\boldsymbol{b}_i$ for subject i, and r is the dimension of $\boldsymbol{b}_i$.
  - $\mu \sim \mathrm{N}(0, 1000)$, $\tau^2 \sim \mathrm{IG}(.001, .001)$.
  - We assume Dirichlet$(4, ..., 4)$ on $\boldsymbol{\pi}^D = (\pi^D_1, ..., \pi^D_{K_D})$.
- In the AFT model for FMP age: we assign independent priors $\mathrm{N}(0, 9/4)$ for all the regression coefficient parameters (i.e., α, θ) and $\mathrm{IG}(.001, .001)$ for the residual variance parameter σ^2.
