# Supplementary material for: Bayesian estimation of associations between identified longitudinal hormone subgroups and age at final menstrual period
Source: BMC Med Res Methodol. 2015 Dec 18;15:106. doi: 10.1186/s12874-015-0101-3 (PMC4683774; doi:10.1186/s12874-015-0101-3)
Supplement: Additional file 3: — Posterior computations through Gibbs sampling. (PDF 100 kb) [file 12874_2015_101_MOESM3_ESM.pdf]

Posterior computations through Gibbs sampling

(1) update for GGMM model for FSH trajectory

- **update** the mean profile class memberships D_i, $i = 1,...,n$: the full conditional posterior distribution $[D_i|\cdot] \sim$ Multinomial$(\tilde{\pi}^D_{i1},...,\tilde{\pi}^D_{iK_D})$, where

$$\tilde{\pi}^D_{id} = \Pr(D_i = d|.) = \frac{\pi^D_d |\Sigma|^{-\frac{1}{2}} \exp\left[-\frac{1}{2}(\boldsymbol{b}_i - \boldsymbol{\beta}_d)'\Sigma_d^{-1}(\boldsymbol{b}_i - \boldsymbol{\beta}_d)\right]}{\sum_{d=1}^{K_D} \pi^D_d |\Sigma|^{-\frac{1}{2}} \exp\left[-\frac{1}{2}(\boldsymbol{b}_i - \boldsymbol{\beta}_d)'\Sigma_d^{-1}(\boldsymbol{b}_i - \boldsymbol{\beta}_d)\right]}.$$

- **update** the mean profile class parameters:
  - **update** $\boldsymbol{\beta}_d = (\beta_{d1},...,\beta_{dL})$:
    Assuming the prior for $\beta_{d1} \sim \mathrm{N}(0,v)$ and first order random walk prior $\beta_{dl} \sim \mathrm{N}(\beta_{d,l-1}, \tau^2_{\beta d})$, $l = 2,...,L$, then the prior for $\boldsymbol{\beta}_d$ can be written as: $\pi(\boldsymbol{\beta}_d) = \left(\frac{1}{\sqrt{2\pi}\tau_{\beta d}}\right)^{L-1} \exp\{-\frac{1}{2}\boldsymbol{\beta}_d^T \boldsymbol{V}\boldsymbol{\beta}_d\}$, where $\boldsymbol{V} = \begin{pmatrix} v^{-1} & \mathbf{0} \\ \mathbf{0} & \mathbf{0} \end{pmatrix} + \boldsymbol{P}^T\boldsymbol{P}/\tau^2_{\beta d}$ and $\boldsymbol{P} = \begin{pmatrix} 1 & -1 & & & \\ & 1 & -1 & & \\ & & \ddots & \ddots & \\ & & & 1 & -1 \end{pmatrix}$ is the $(L-1)\times L$ penalty matrix. Then the full conditional posterior density for $\boldsymbol{\beta}_d$ for $d = 1,...,K_D$ is $[\boldsymbol{\beta}_d|.] \sim$ MVN$\left(\tilde{\boldsymbol{\nu}}_d, \tilde{\boldsymbol{V}}_d\right)$

$$\tilde{\boldsymbol{\nu}}_d = \left[\boldsymbol{V} + \boldsymbol{\Sigma}^{-1}\sum_{i=1}^{n}\mathrm{I}(D_i = d)\right]^{-1}\left[\boldsymbol{\Sigma}^{-1}\sum_{i=1}^{n}\mathrm{I}(D_i = d)\boldsymbol{b}_i\right]$$
$$\tilde{\boldsymbol{V}}_d = \left[\boldsymbol{V} + \boldsymbol{\Sigma}^{-1}\sum_{i=1}^{n}\mathrm{I}(D_i = d)\right]^{-1}.$$

  - **update** $\boldsymbol{\Sigma}$: Assuming the prior for $\boldsymbol{\Sigma} \overset{ind}{\sim}$ Inverse-Wishart$(m, \boldsymbol{\Lambda})$, where m and $\boldsymbol{\Lambda}$ are the degrees of freedom and scale matrix, respectively, then the full conditional posterior density is, $[\boldsymbol{\Sigma}|\cdot] \sim$ Inverse-Wishart$\left(\tilde{m}_d, \tilde{\boldsymbol{\Lambda}}_d\right)$ where

$$\tilde{m}_d = m + n$$
$$\tilde{\boldsymbol{\Lambda}}_d = \left[\Lambda + \sum_{i=1}^{n}\left(\boldsymbol{b}_i - \boldsymbol{\beta}_{D_i}\right)\left(\boldsymbol{b}_i - \boldsymbol{\beta}_{D_i}\right)'\right].$$

  - **update** $\tau^2_{\beta d}$: assuming $\tau^2_{\beta d} \sim$ Inverse-Gamma(v, e), where v and e are the shape and rate parameters, then the full conditional posterior distribution is

$$\left[\tau^2_{\beta d}|\cdot\right] \sim \text{Inverse-Gamma}\left(v + \frac{L-1}{2}, e + \frac{1}{2}\boldsymbol{\beta}_d^T\boldsymbol{P}\boldsymbol{\beta}_d\right),$$

    where L is the number of B spline basis functions.

- **update** the mixing proportion $\{\pi_d^D\}_d$: assuming $\left[\{\pi_d^D\}_d\right] \sim \text{Dirichlet}(e_1^D, ..., e_{K_D}^D)$ then the full conditional posterior distribution is

$$[\{\pi_d^D\}_d|\cdot] \sim \text{Dirichlet}(\{e_d^D + \sum_{i=1}^{n} \text{I}(D_i = d)\}_d).$$

- **update** the variance parameters:
  - **update** μ: assuming the prior for $\mu \overset{ind}{\sim} \text{N}(a,b)$, then the full conditional posterior distribution is, $[\mu|\cdot] \sim \text{N}\left(\tilde{a},\tilde{b}\right)$ where

$$\tilde{a} = \frac{\sum_{i=1}^{n} \log\sigma_i^2/\tau^2 + a/b}{1/b + \sum_{i=1}^{n} /\tau^2}$$

$$\tilde{b} = \left(1/b + \sum_{i=1}^{n} /\tau^2\right)^{-1}$$

  - **update** τ^2: assuming $\tau^2 \sim \text{Inverse-Gamma}(v,e)$, then the full conditional posterior distribution is $\left[\tau^2|\cdot\right] \sim \text{Inverse-Gamma}\left(v + \frac{n}{2}, e + \sum_{i=1}^{n} \frac{1}{2}\left(\log\sigma_i^2 - \mu\right)^2\right)$.

- **update** the random effects $\boldsymbol{b}_i$, $i = 1,...,n$ the full conditional posterior distribution is $\boldsymbol{b}_i$ $[\boldsymbol{b}_i|.] \sim \text{MVN}\left(\tilde{\beta}_i, \tilde{\Sigma}_i\right)$, where

$$\tilde{\Sigma}_{id} = \left[\Sigma^{-1} + \frac{1}{\sigma_i^2}\sum_{j=1}^{n_i} m_{ij}\phi_{ij}\phi_{ij}'\right]^{-1}$$

$$\tilde{\beta}_i = \tilde{\Sigma}_{id}\left[\Sigma^{-1}\beta_{D_i} + \frac{1}{\sigma_i^2}\sum_{j=1}^{n_i} y_{ij}m_{ij}\phi_{ij}\right],$$

where, $\phi_{ij} = (\phi_1(t_{ij}), \cdots, \phi_L(t_{ij}))^T$ a vector of B spline basis functions evaluated at time t_{ij} such that $\mu_i(t_{ij}) = \boldsymbol{b}_i'\phi_{ij}$ where $y_{ij} \sim \text{N}\left(\mu_i(t_{ij}), \sigma_i^2/m_{ij}\right)$.

- **update** the variances σ_i^2, $i = 1,...,n$

$$\pi(\sigma_i^2|.) \propto (\sigma_i^2)^{-\frac{n_i}{2}-1}\exp\left[-\frac{\left(\log\sigma_i^2 - \mu\right)^2}{2\tau^2} - \frac{1}{2\sigma_i^2}\sum_{j=1}^{n_i} m_{ij}\left(y_{ij} - \boldsymbol{b}_i'\phi_{ij}\right)^2\right]$$

  Since there is no closed form of the full conditional posterior density, the draws for σ_i^2, $i = 1,...,n$ at each iteration of the Gibbs sampling are obtained using the inverse cumulative distribution sampling method.

- **update** m_{ij}, $j = 1,...,n_i$, given that $m_{ij} \sim \text{Gamma}(v/2, v/2)$ where $v/2$ and $v/2$ are the shape and rate parameter in Gamma distribution, then the full conditional posterior distribution for m_{ij} is $m_{ij} \sim \text{Gamma}\left(\frac{v+1}{2}, \frac{1}{2}\left(\frac{\left(y_{ij}-\mu(t_{ij})\right)^2}{\sigma_i^2} + v\right)\right)$.

(2) update for the AFT model for FMP age:
We re-express the different AFT models given in Table 1 by the general form: $\log(T_i - 40) = \boldsymbol{u}_i^T \boldsymbol{\rho} + \varepsilon_i$, where $\boldsymbol{u}_i$ is a vector of constant 1 and the covariates in the AFT models (including the extracted FSH features and other baseline covariates) and $\boldsymbol{\rho}$ is the coefficient vector.

- **update** censored age at FMP is drawn from a conditional distribution given the fact that the age at FMP is larger than the censoring age C_i: let s_i be a random draw from the truncated normal distribution $\mathrm{N}\left(\boldsymbol{u}_i^T \boldsymbol{\rho}, \sigma^2\right) \mathrm{I}(C_i, \infty)$, and T_i can be obtained as $T_i = \exp(s_i + 40)$.

- **update** $\boldsymbol{\rho}$: assuming the prior $\boldsymbol{\rho} \sim \mathrm{MVN}(\mathbf{0}, \boldsymbol{V})$, then the full conditional posterior density for $\boldsymbol{\rho}$ is $[\boldsymbol{\rho}|\cdot] \sim \mathrm{MVN}\left(\tilde{\boldsymbol{\nu}}, \tilde{\boldsymbol{V}}\right)$ where

$$\tilde{\boldsymbol{\nu}} = \left[\boldsymbol{V}^{-1}\sigma^2 + \sum_{i=1}^{n} \boldsymbol{u}_i \boldsymbol{u}_i'\right]^{-1} \sum_{i=1}^{n} [\boldsymbol{u}_i \log(T_i - 40)]$$
$$\tilde{\boldsymbol{V}} = \left[\boldsymbol{V}^{-1} + \sigma^{-2} \sum_{i=1}^{n} \boldsymbol{u}_i \boldsymbol{u}_i'\right]^{-1},$$

- **update** σ^2: assuming $\sigma^2 \sim \mathrm{IG}(f, g)$, where f and g are the shape and rate parameters, then the full conditional posterior distribution is $\left[\sigma^2|\cdot\right] \sim \mathrm{IG}\left(f + \frac{n}{2}, g + \sum_{i=1}^{n} (\log(T_i - 40) - \boldsymbol{u}_i^T \boldsymbol{\rho})^2\right)$.
